# HELQ and EGR3 expression correlate with IGHV mutation status and prognosis in chronic lymphocytic leukemia

**DOI:** 10.1186/s12967-021-02708-6

**Published:** 2021-01-23

**Authors:** Chao Guo, Ya-yue Gao, Qian-qian Ju, Chun-xia Zhang, Ming Gong, Zhen-ling Li

**Affiliations:** Department of Hematology, China-Japan Friendship Hospital, Yinghua East Street, Beijing, 100029 China

**Keywords:** Chronic lymphocytic leukemia, Survival analysis, Gene expression profile, IGHV mutation

## Abstract

**Background:**

IGHV mutation status is a crucial prognostic biomarker for CLL. In the present study, we investigated the transcriptomic signatures associating with IGHV mutation status and CLL prognosis.

**Methods:**

The co-expression modules and hub genes correlating with IGHV status, were identified using the GSE28654, by ‘WGCNA’ package and R software (version 4.0.2). The over-representation analysis was performed to reveal enriched cell pathways for genes of correlating modules. Then 9 external cohorts were used to validate the correlation of hub genes expression with IGHV status or clinical features (treatment response, transformation to Richter syndrome, etc.). Moreover, to elucidate the significance of hub genes on disease course and prognosis of CLL patients, the Kaplan–Meier analysis for the OS and TTFT of were performed between subgroups dichotomized by the median expression value of individual hub genes.

**Results:**

2 co-expression modules and 9 hub genes ((FCRL1/FCRL2/HELQ/EGR3LPL/LDOC1/ZNF667/SOWAHC/SEPTIN10) correlating with IGHV status were identified by WGCNA, and validated by external datasets. The modules were found to be enriched in NF-kappaB, HIF-1 and other important pathways, involving cell proliferation and apoptosis. The expression of hub genes was revealed to be significantly different, not only between CLL and normal B cell, but also between various types of lymphoid neoplasms. HELQ expression was found to be related with response of immunochemotherapy treatment significantly (p = 0.0413), while HELQ and ZNF667 were expressed differently between stable CLL and Richter syndrome patients (p < 0.0001 and p = 0.0278, respectively). By survival analysis of subgroups, EGR3 expression was indicated to be significantly associated with TTFT by 2 independent cohorts (GSE39671, p = 0.0311; GSE22762, p = 0.0135). While the expression of HELQ and EGR3 was found to be associated with OS (p = 0.0291 and 0.0114 respectively).The Kras, Hedgehog and IL6-JAK-STAT3 pathways were found to be associating with the expression of hub genes, resulting from GSEA.

**Conclusions:**

The expression of HELQ and EGR3 were correlated with IGHV mutation status in CLL patients. Additionally, the expression of HELQ/EGR3 were prognostic markers for CLL associating with targetable cell signaling pathways.

## Background

CLL (chronic lymphocytic leukemia) is characterized by uncontrolled proliferation of monoclonal B cells, and resistance to cell apoptosis. CLL is the most prevalent adult leukemia in Europe and America. The age-adjusted incidence in United States is 4.1 per 100,000 inhabitants, with 4500 estimated deaths [1]. The diagnosis requires monoclonal B cells count more than 5 $\times$ 10^3^/L in peripheral blood, with characteristic morphology and immunophenotype (typically positive for CD5, CD23, CD19, CD20 and CD200). The disease course of CLL is heterogenous, and the treatment was initiated only in patients with advancing or symptomatic disease. The immunochemotherapy, including anti-CD20 monoclonal antibody and cytotoxic agents (fludarabine and cyclophosphamide, etc.) was the traditional choice. While novel agents, including BTK (Bruton’s Tyrosine Kinase) inhibitor (ibrutinib, zanubrutinib) and BCL2 (B cell lymphoma 2) inhibitor (venetoclax), have greatly improved the survival of CLL patients.

Due to the high heterogeneity of CLL, several risk score systems have bene established, among which 2 most widely accepted systems are Rai and Binet risk stratification developed about 40 years ago [1]. These clinical staging systems seemed insufficient for clinical practice, due to the rapid progress of treatment. More molecular and cytogenetic markers were included in the current scoring system, such as CLL International Prognosis Index (CLL-IPI) [2], which included TP53 gene mutation/deletion, IGHV mutation status, serum beta2 microglobulin, clinical stage and age. The IGHV (immunoglobulin heavy-chain variable region) gene mutation status is demonstrated to be a pivotal prognostic marker for CLL. The U-CLL (unmutated CLL) patients have shorter lymphocyte doubling time and higher expression of CD38, and correlated more aggressive disease course with shorter TTFT (time to first treatment) in comparison with that of M-CLL (mutated CLL) patients [3–5]. Moreover, the unmutated IGHV status predicts unfavorable OS (overall survival) for CLL patients receiving immunochemotherapy [6]. However, the expression signature associating with IGHV mutation has been rarely been investigated.

**Fig. 1:**
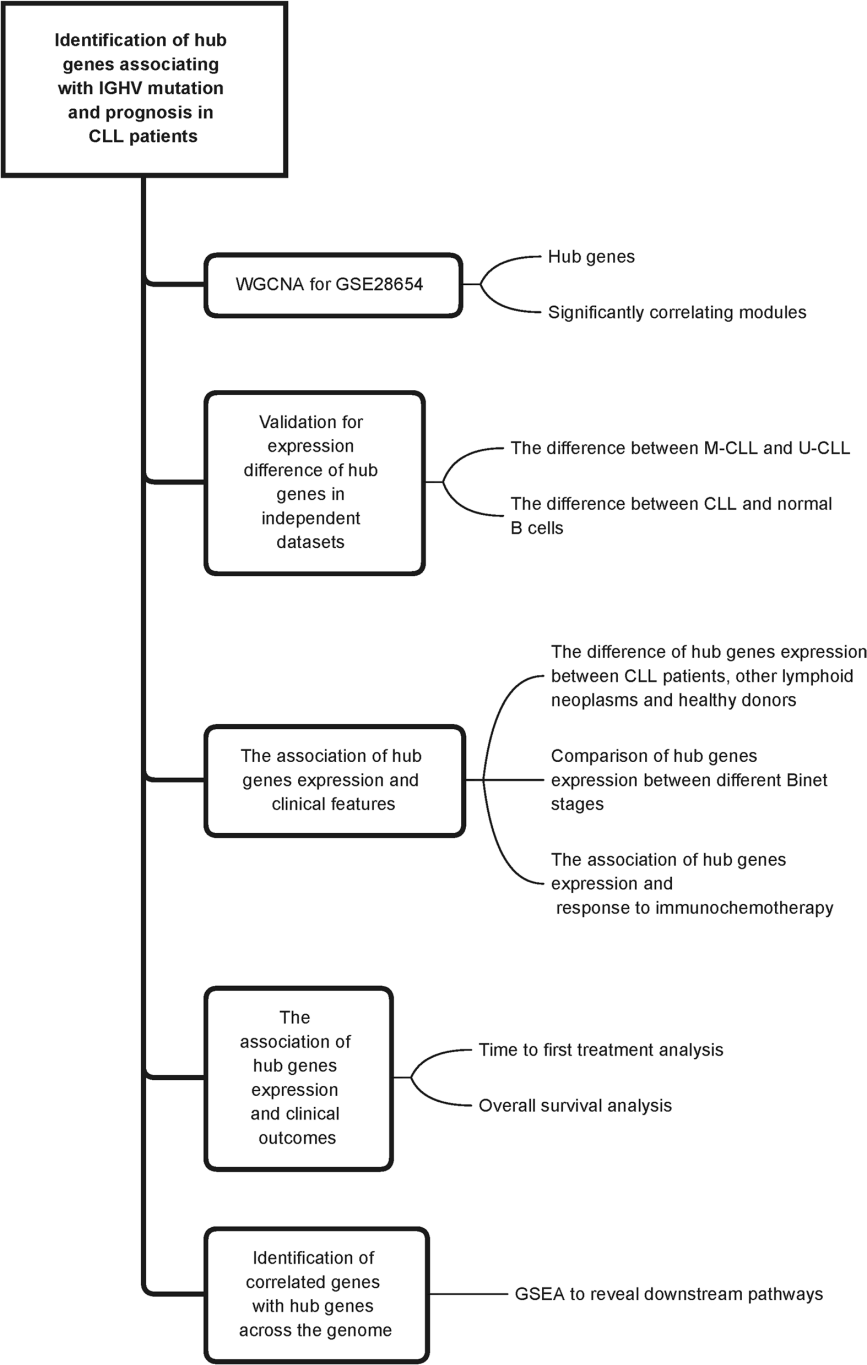
The overall flow chart of our present study

The transcriptomic analysis based on microarray and RNAseq methods, have preliminarily revealed the expression profiles and altered signaling pathways for CLL, which have provided potential biomarkers and therapeutic targets [7–10]. By WGCNA (weighted gene co-expression network analysis), the hub genes and co-expression modules associating with IGHV status, were identified and validated. The expression signature of hub genes were also associated with clinical features (response to immunochemotherapy and Richter transformation) and clinical outcomes (OS and TTFT). The flowchart for the overall design of this work was shown in Fig. 1. Our work revealed the transcriptomic signature characterized by co-expression modules, and provided insights and rationales to utilize HELQ/EGR3 expression as prognostic markers for CLL.

## Methods

### Data source

The expression matrix and clinical/genetic data was downloaded from GEO database repository (https://www.ncbi.nlm.nih.gov/gds/). The details of GEO datasets used in this study were summarized in Table 1 [7–9, 11–16]. Since the WGCNA required representative samples and expression information of full-scale genome. Among the 6 independent datasets including IGHV mutation information (Table 1), GSE38611/40570/51529 included only early stage patients (Binet A). And GSE69034 used total lymphocytes instead of purified B cells, which may lead to bias to transcriptomic analysis. GSE9992 was performed in GPL96 platform, which were only able to detect 12,402 genes and resulted in missing value for many genes. Therefore, GSE28654 was selected for WGCNA analysis, which avoided abovementioned problems. The last access to the GEO database is on 2020.10.15.

**Table 1:** The summary of GEO datasets used in the present study

GEO accession	Number of samples	patients subgroup	Application in this study
GSE28654 [7]	89	61 M-CLL and 28 U-CLL	WGCNA to reveal hub genes correlating with IGHV status
GSE38611 [8]	136	76 M-CLL and 60 U-CLL	Validation for the correlation of hub gene expression with IGHV status
GSE40570 [11]	159	96 M-CLL vs 63 U-CLL	Validation for the correlation of hub gene expression with IGHV status
GSE51529 [9]	229	131 M-CLL and 85 U-CLL	Validation for the correlation of hub gene expression with IGHV status
GSE69034	144	86 M-CLL and 58 U-CLL	Validation for the correlation of hub gene expression with IGHV status
GSE9992 [12]	60	24 M-CLL and 36 U-CLL	Validation for the correlation of hub gene expression with IGHV status
GSE50006	210	188 CLL and 32 healthy donors	Validation for expression difference of hub genes between CLL and healthy dornors
GSE32018 [23]	127	17 CLL, 23 FL, 22 DLBCL, 24 MCL, 15 MALT-MZL, 13 NMZL and 13 normal lymphoid tissues	Validation for expression difference of hub genes between various type of lymphoid neoplasms
GSE58211 [13]	300	29 Binet stage A, 179 Binet stage B and 92 Binet stage C CLL patients	Validation for expression difference of hub genes between different stages of CLL
GSE10138 [14]	68	32 progressive and 36 stable CLL patients	Validation for the correlation of hub gene expression with treatment response in CLL patients
GSE103265	19	8 CLL and 11 Richter syndrome	Validation for expression difference of hub genes between stable CLL and Richter syndrome patients
GSE39671 [15]	130	130 CLL	survival analysis of TTFT stratificated by hub gene expression
GSE22762 [16]	107	107 CLL	survival analysis of TTFT/OS stratificated by hub gene expression

### Weighted gene co-expression network analysis

The weighted gene co-expression network was based on the expression data of the whole genome, by ‘WGCNA’ package [17] and R software (version 4.0.2). The outliers among samples were detected by hierarchical clustering by average link. The soft threshold power was defined as the minimal beta value which set the scale-free R^2^ > 0.85. Then inter-gene correlating coefficients were calculated by Pearson’s method, which constructed the matrix of gene adjacency and turned into TOM (topological overlap matrix) sequentially. The minimal size of co-expression modules was limited to 30 genes. Genes in the whole genome were classified into co-expression modules according to TOM-based dissimilarity by average linkage hierarchical clustering method. The first principal component of expression matrix is set as module eigengenes. The module membership of individual genes was defined as the correlation coefficients between gene expression and eigengene of the module. The gene significance of individual genes was defined as the correlation coefficients between gene expression and IGHV status. The modules were identified as targets, with the highest Pearson’s coefficient with IGHV status. Within target modules, the hub genes were defined as gene module membership ≥ 0.8, weighted q value < 0.01, and gene significance ≥ 0.2.

Additionally, PPI (protein–protein interaction) network was established for target modules by STRING database (https://string-db.org/) based on the previous evidence and experiments. The genes of target modules were mapped into STRING, and the criteria of extracting PPI pairs was that confidence ≥ 0.4.

### Over-representation analysis for the correlated modules

The ORA (over-representation analysis) was performed for genes in the ‘black’ and ‘purple’ modules using hypergeometric distribution method. The analysis was performed by CPDB online tools [18] (http://cpdb.molgen.mpg.de/) based on GO (gene ontology) database [19, 20] and KEGG (Kyoto Encyclopedia of Genes and Genomes) database [21, 22] respectively. P value < 0.05 was set as the criteria of enriched pathways.

### Validation of hub genes expression signature

To validate the association of hub genes and IGHV mutation status, the expression data was normalized by ‘limma’ package in R software (version 4.0.2), gene expression was compared between M-CLL and U-CLL groups using 6 independent datasets (GSE38611, GSE40570, GSE51529, GSE69034 and GSE9992). Due to the EGR3 expression data was missing in GSE9992, only HELQ/ZNF667/SOWAHC were analyzed for this dataset. Then the comparison of hub genes expression was performed across different lymphoid neoplasms (CLL/FL/DLBCL/MALT-MZL/NMZL/MCL) and normal lymphoid tissues, using GSE50006 and GSE32018 [23].

### The association of hub genes and clinical features of CLL patients

The expression level of hub genes was extracted from the whole transcriptomic datasets. GSE58211 [13] was used to compare the hub genes expression between different Binet stages. GSE10138 dataset was utilized for comparison of hub genes expression between response groups to immunochemotherapy. Moreover, the hub genes transcription was compared between Richter transformed and non-transformed CLL patients of GSE103265.

### Survival analysis for hub genes expression in CLL

The GSE39761 provided individual TTFT data for 130 untreated CLL patients. Meanwhile, the GSE22762 consisted of individual TTFT data of 70 CLL patients, and OS data for 107 CLL patients. The cohorts were dichotomized into low and high expression groups by the median value of hub genes expression.

### Genome-wide gene expression profile associating with hub genes

Due to the prognostic significance of HELQ and EGR3 genes, the expression correlation analysis was performed to uncover the associating genes. We calculated Pearson’s coefficients for individual genes of the whole genome. R software (version 4.0.2) and ‘stats’ package was utilized for the calculation. The criteria of associating genes were defined as |R^2^|> 0.45 and p value < 0.05. Then, The GSEA was performed to access the enrichment of HELQ/EGR3 associating genes on signaling pathways, according to MSigDB database [25–27] (http://software.broadinstitute.org/gsea/msigdb). The significantly enriched pathways were selected with |NES (normalized enrichment score)|> 1 and q value < 0.05.

### Statistical analysis

The expression data was normalized by ‘normalizeBetweenArrays’ function in ‘limma’ package from R software (version 4.0.2). The unpaired t test was utilized to compare the normalized continuous variables between 2 subgroups. In the situation of comparing between more than 2 groups, the ordinary one-way ANOVA test was used. The logrank test was utilized to test the survival difference between subgroups, using p < 0.05 as cut-off value.

## Results

### The results of WGCNA

**Fig. 2:**
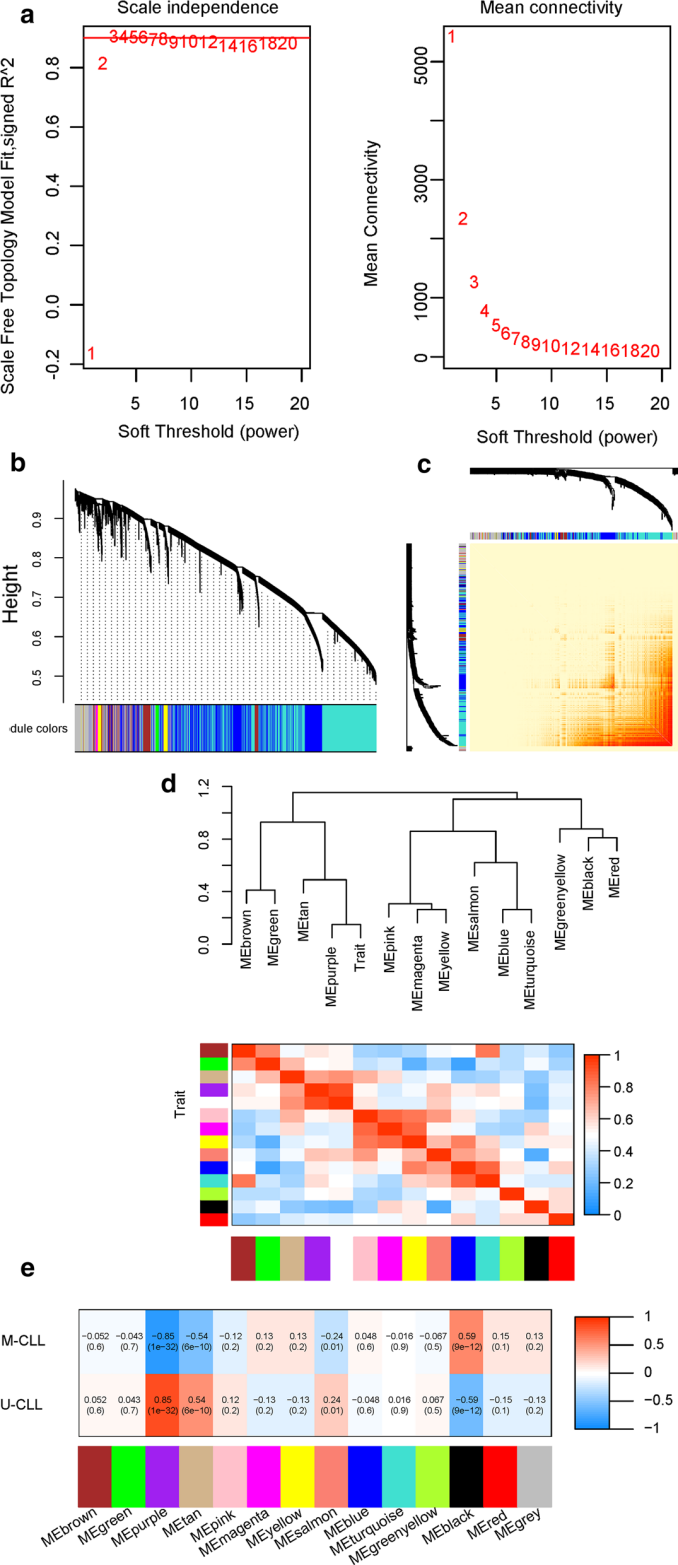
**a** The scale independence (the left plot) and mean connectivity (the right plot) corresponding to different soft-thresholding values. **b** The cluster dendrogram (the upper part) and the co-expression modules (the lower part) generated by average linkage hierarchical clustering method. the branches of the dendrogram represent individual genes. The height indicates the Euclidean distance. Each module that contains weighted co-expressed genes, is displayed with a distinct color. **c** The heatmap of topological overlap using 400 randomly selected genes. The genes are divided into different colors (modules), shown under the cluster dendrogram. **d** The heatmap of module eigengene adjacency, which stands for the relationship between distinct co-expression modules. **e** The module-trait relationship plotter. All modules (colors) are displayed on the longitudinal axis, while all prognostic markers are displayed on the transverse axis. Each cell contains R^2^ and p value of correlations between the modules and prognostic markers by Spearman’s method. The gradient color of each cell corresponds to the R^2^ (red = 1, blue =  − 1)

The GSE28654 cohort included 61 M-CLL, 28 U-CLL and 23 CLL with undetermined IGHV status (excluded from WGCNA) [7]. The median age at diagnosis was 61 years old, and the majority of the cohort were in early stages (104 Binet stage A and 8 Binet stage B patients) and untreated (78 untreated vs 34 treated patients) [7]. No outliers were detected by hierarchically clustering with average distance (Additional file 1: Fig. S1). We selected 3 as the soft threshold power according to Fig. 2a. The maximal dissimilarity was set as 15% for merging similar modules, resulting in a total of 14 co-expression modules. The topological overlap degree of individual modules was shown in Fig. 2c, which was generated in the form of topological overlap heatmap after grouping 4 hundred randomly selected genes into modules. Additionally, the eigengene adjacency heatmap demonstrated the relationship between modules (Fig. 2d). The Pearson’s coefficients and p values, generated from the correlation analysis between individual module eigengenes and IGHV status, were shown in Fig. 2e. The correlation between the ‘black’ module and M-CLL was the most significant (R^2^ = 0.59, p = 9e−12). Whereas the ‘purple’ module was the most correlated with U-CLL (R^2^ = 0.85, p = 1e−32). 4 genes (FCRL1/FCRL2/HELQ/EGR3) of the ‘black’ module, and 5 genes (LPL/LDOC1/ZNF667/SOWAHC/SEPTIN10) of the ‘purple’ module were identified as hub genes.

To our best knowledge, HELQ/EGR3/ZNF667/SOWAHC were not described to be relevant with CLL previously. As an important regulation factor of DNA repair pathway, HELQ (Helicase POLQ-like protein) expression is demonstrated as an indicator of resistance to platinum based chemotherapy in epithelial ovarian cancer [28]. Hui Cheng et al. demonstrate that EGR3 (Early growth response protein 3) is a strong limiting factor for potential of hematopoietic stem cell proliferation [29]. Recently, the under-expression of EGR3 is demonstrated to be an independent risk factor for metastatic prostate cancer [30]. ZNF667, encoding Zinc finger protein 667, may involve in transcription regulation, the aberrantly hypermethylation of which promoted progression of laryngeal and esophageal squamous cell carcinoma [31, 32]. SOWAHC, encoding Ankyrin repeat domain-containing protein SOWAHC, is demonstrated to be prognostic in bladder cancer [33] and lung squamous cell carcinoma [34]. The 4 genes were selected as target genes in the following analysis, according to prognostic value in abovementioned studies and correlation with IGHV status in WGCNA. Moreover, the genes of ‘black’ and ‘purple’ modules were mapped into STRING tools, to establish the PPI network (Additional file 2: Fig. S2).

### The results of ORA for co-expression modules

**Fig. 3:**
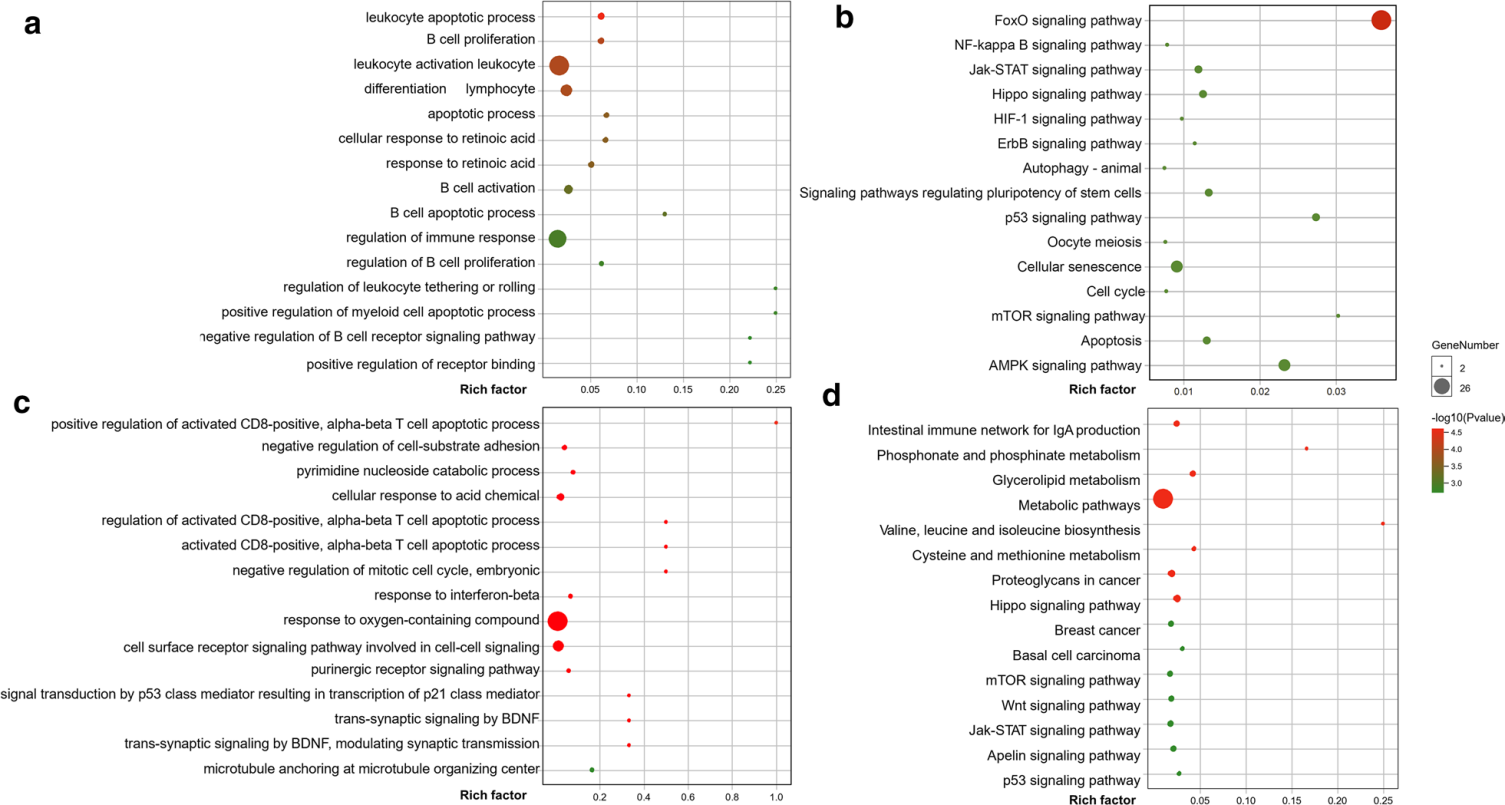
**a**, **b** The results ORA for the ‘black’ module (GO/KEGG). **a**, **b** The results ORA for the ‘purple’ module (GO/KEGG). The X-axis represented the rich factor, and the diameter of dots indicated gene number involved in the gene set. Additionally, the color of dots correlated with the −log10(p value)

For the ‘black’ module, the genes were mainly enriched in biological processes involving with B cells, like B cell activation, B cell proliferation, B cell apoptotic process, negative regulation of B cell receptor signaling pathway, etc. (Fig. 3a). And the molecular functions of the genes were enriched in binding, catalytic activity, etc. whereas the products of genes were predominantly located in cell, membrane, etc. Based on KEGG database, the genes were predominantly enriched in NF-kappa B signaling, JAK-STAT signaling, p53 signaling pathway, etc. (Fig. 3b).

For the ‘purple’ module, the genes were mainly enriched in biological processes, like regulation of activated CD8-positive alpha–beta T cell apoptotic process, signal transduction by p53 class mediator resulting in transcription of p21 class mediator, etc. (Fig. 3c). And the molecular functions of the genes were enriched in transport activity, catalytic activity, etc. whereas the products of genes were predominantly located in cell, membrane, etc. Based on KEGG database, the genes were predominantly enriched in mTOR signaling, Wnt signaling, p53 signaling pathway, etc. (Fig. 3d).

### Validation of hub genes by additional independent CLL cohorts

**Fig. 4:**
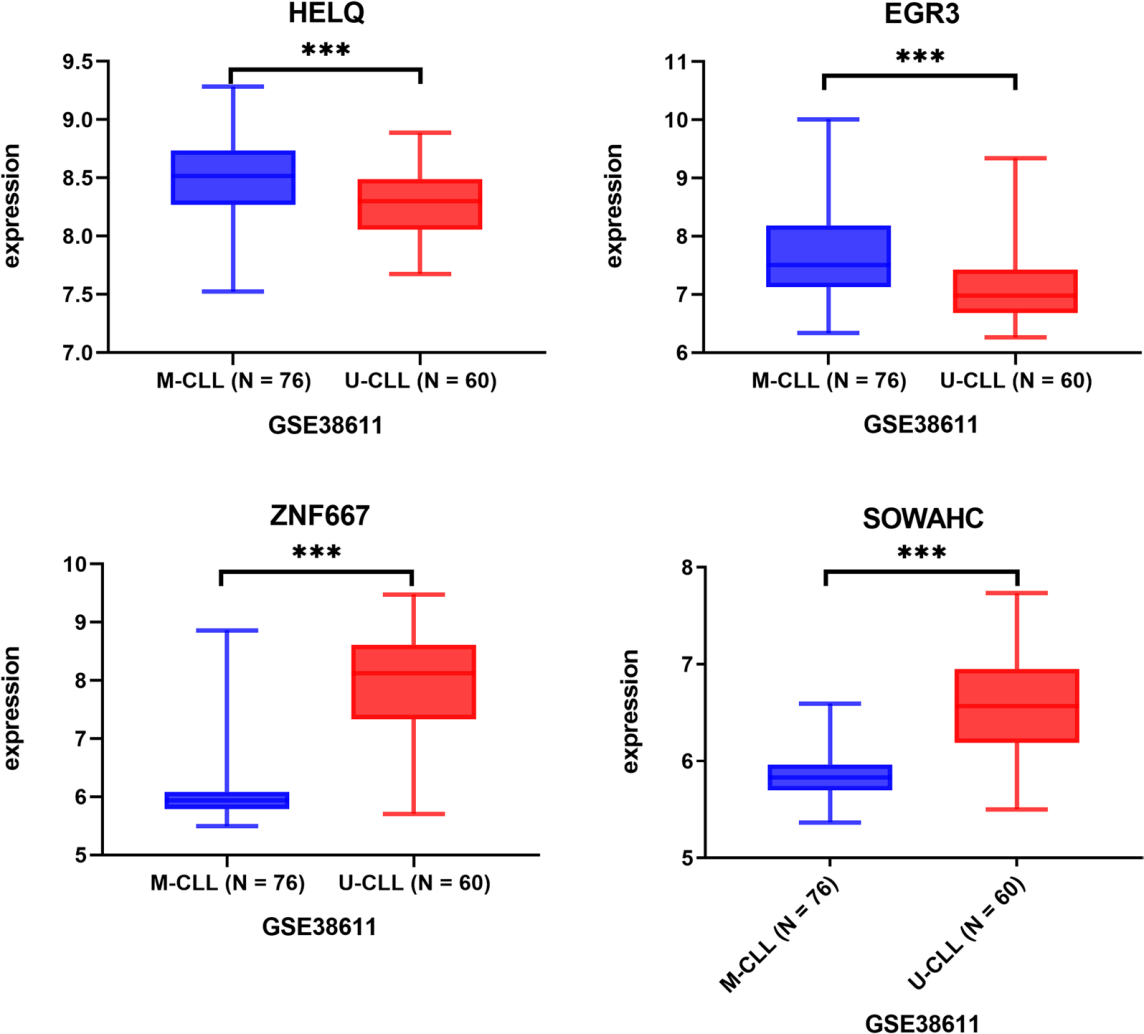
The comparison of hub genes expression between M-CLL and U-CLL based on GSE38611. ***p < 0.0001

**Fig. 5:**
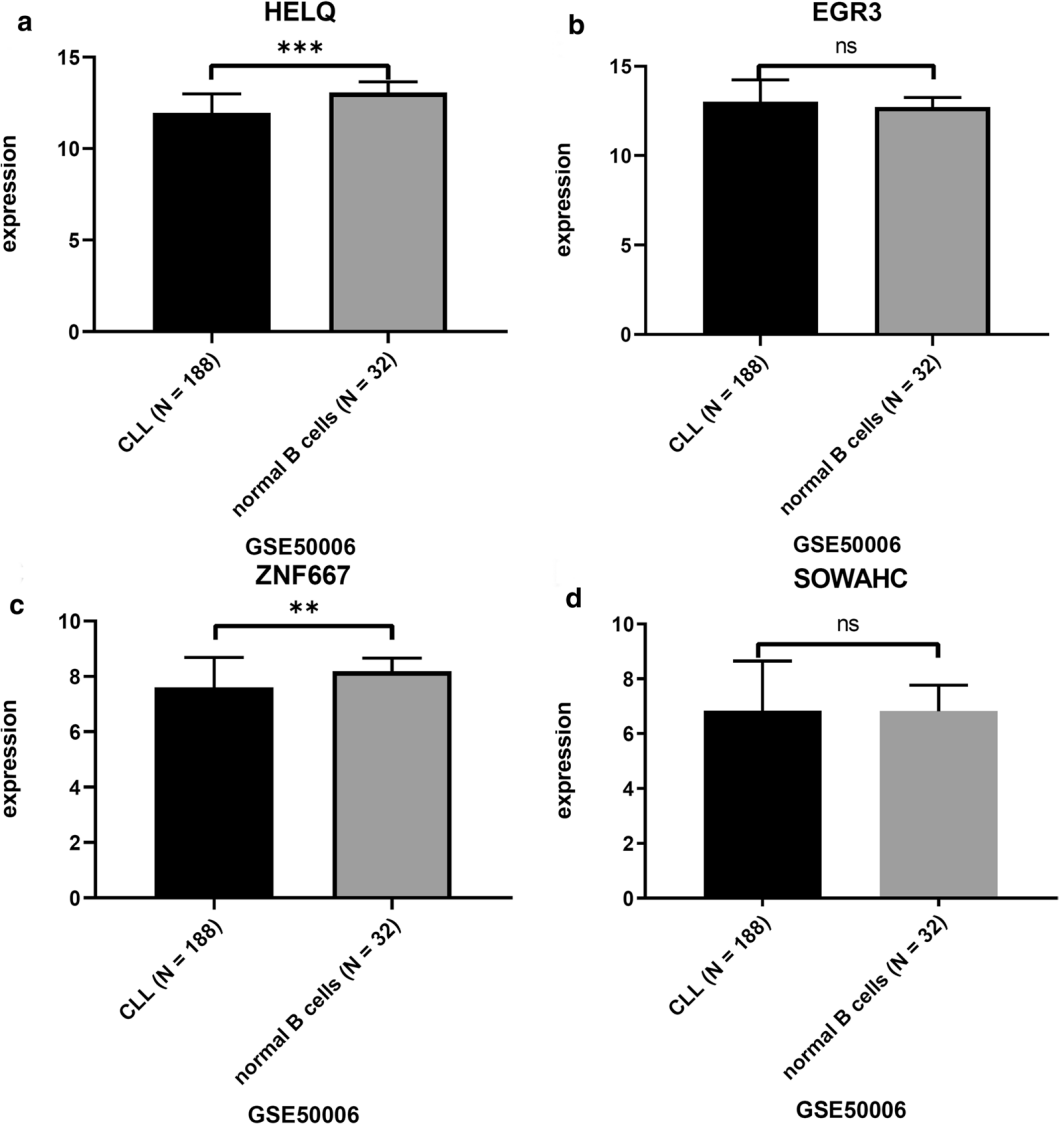
The comparison of hub genes expression between CLL and normal B cells based on GSE50006. ***p < 0.0001. **p < 0.01. ns, p > 0.05

**Fig. 6:**
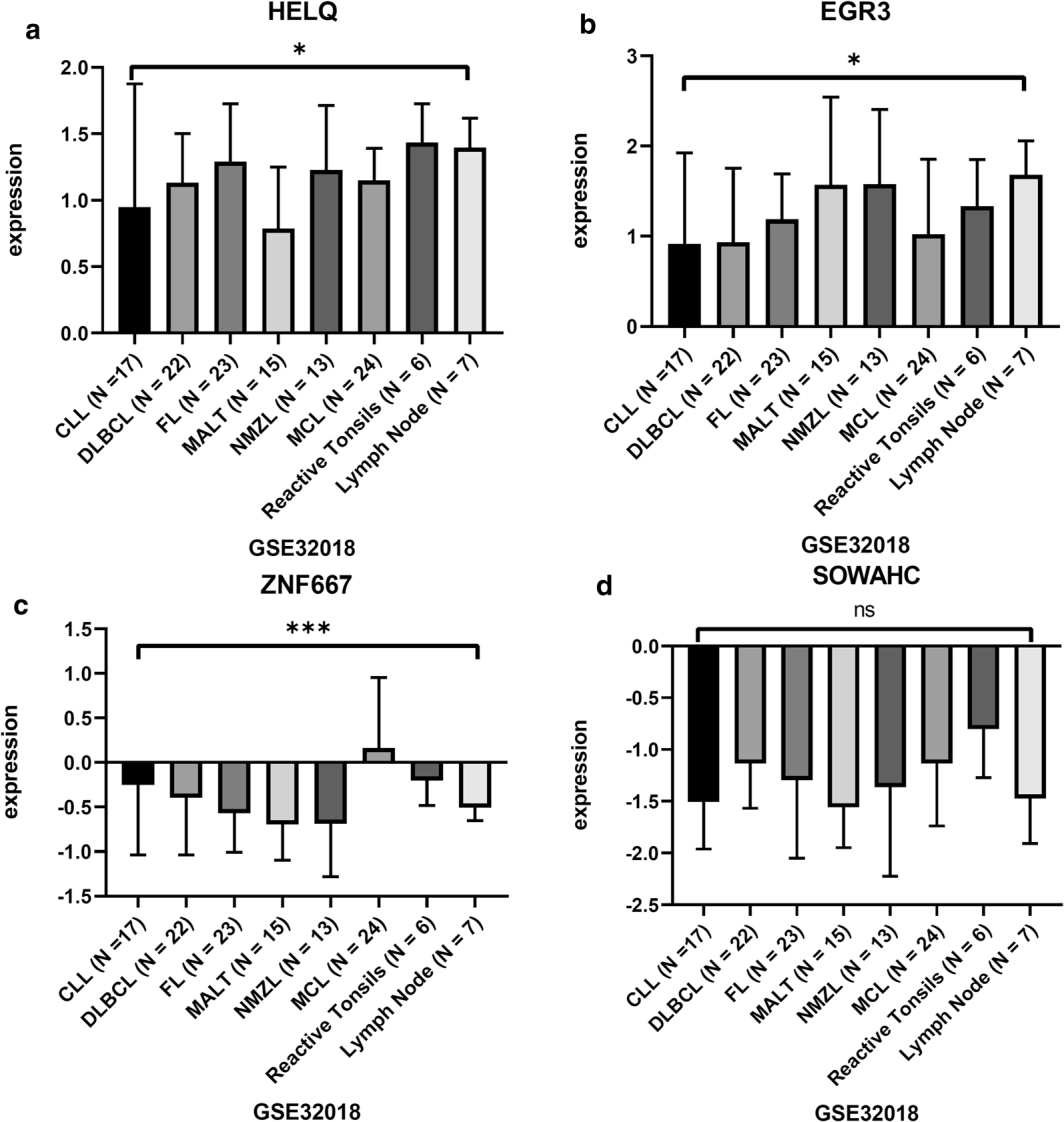
The comparison of hub genes expression between different types of lymphoid neoplasms based on GSE32018. ***p < 0.0001. *p < 0.05. ns, p > 0.05

The comparison of hub genes expression level between M-CLL and U-CLL patients based on GSE38211 was shown in Fig. 4, in which the HELQ and EGR3 expression was significantly higher in M-CLL group than that in U-CLL group. While ZNF667 and SOWAHC expression was significantly lower in M-CLL than that in U-CLL group. Similar results were calculated and obtained from other independent datasets (Table 2). The expression of HELQ, and ZNF667 was also significantly different between CLL leukemic cells and normal B cells (Fig. 5, Table 2). The expression was significantly differential across various types of lymphoid neoplasms for HELQ/EGR3/ZNF667 (Fig. 6, Table 2).

**Table 2:** The comparison of hub genes (HELQ/EGR3/ZNF667/SOWAHC) expression indicated significant difference between M-CLL and U-CLL using unpaired t-test. Since the HELQ expression was not included in the platform of GSE9992, the p-value was not calculated

	GSE38611 (76M-CLL vs 60 U-CLL)	GSE40570 (96M-CLL vs 63 U-CLL)	GSE51529 (131M-CLL vs 85 U-CLL)	GSE69034 (86M-CLL vs 58 U-CLL)	GSE9992 (24M-CLL vs 36 U-CLL)	GSE50006 (188 CLL vs 32 healthy donors)	GSE32018 (17 CLL, 23 FL, 22 DLBCL, 24 MCL, 15 MALT-MZL, 13 NMZL,13 lymphoid tissues
	p value (t test)	p value (t test)	p value (t test)	p value (t test)	p value (t test)	p value (t test)	p value (one-way ANOVA test)
HELQ	< 0.0001	0.0004	< 0.0001	0.748	NA	< 0.0001	0.0236
EGR3	0.0003	< 0.0001	< 0.0001	< 0.0001	0.0015	0.1509	0.0493
ZNF667	< 0.0001	< 0.0001	< 0.0001	< 0.0001	< 0.0001	0.0034	0.0003
SOWAHC	< 0.0001	< 0.0001	< 0.0001	< 0.0001	< 0.0001	0.9806	0.0729

### Expression of hub genes was associated with treatment response and Richter transformation of CLL patients

**Fig. 7:**
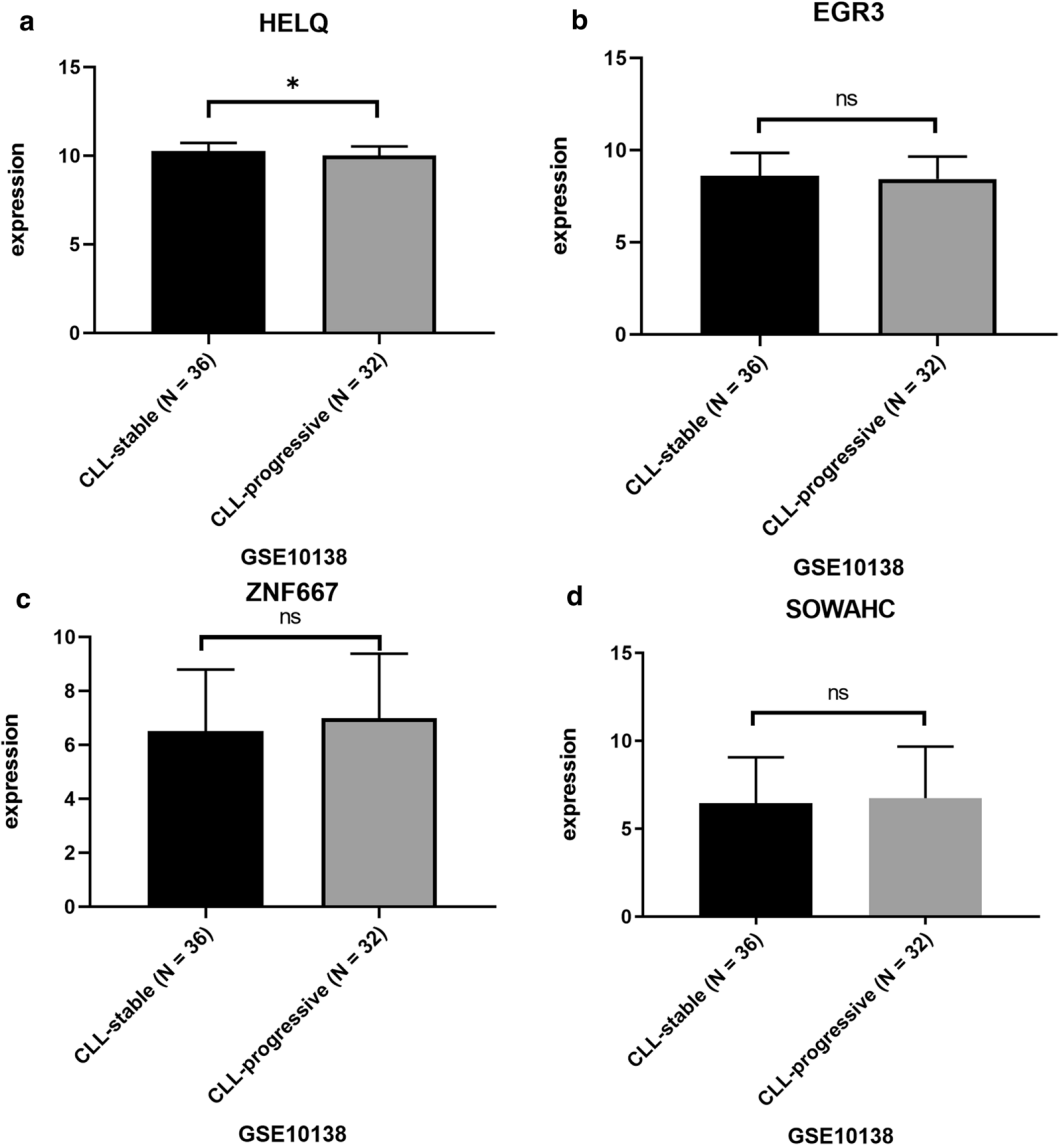
The comparison of hub genes expression between CLL-stable and CLL-progressive group after immunochemotherapy based on GSE10138. *p < 0.05. ns, p > 0.05

**Fig. 8:**
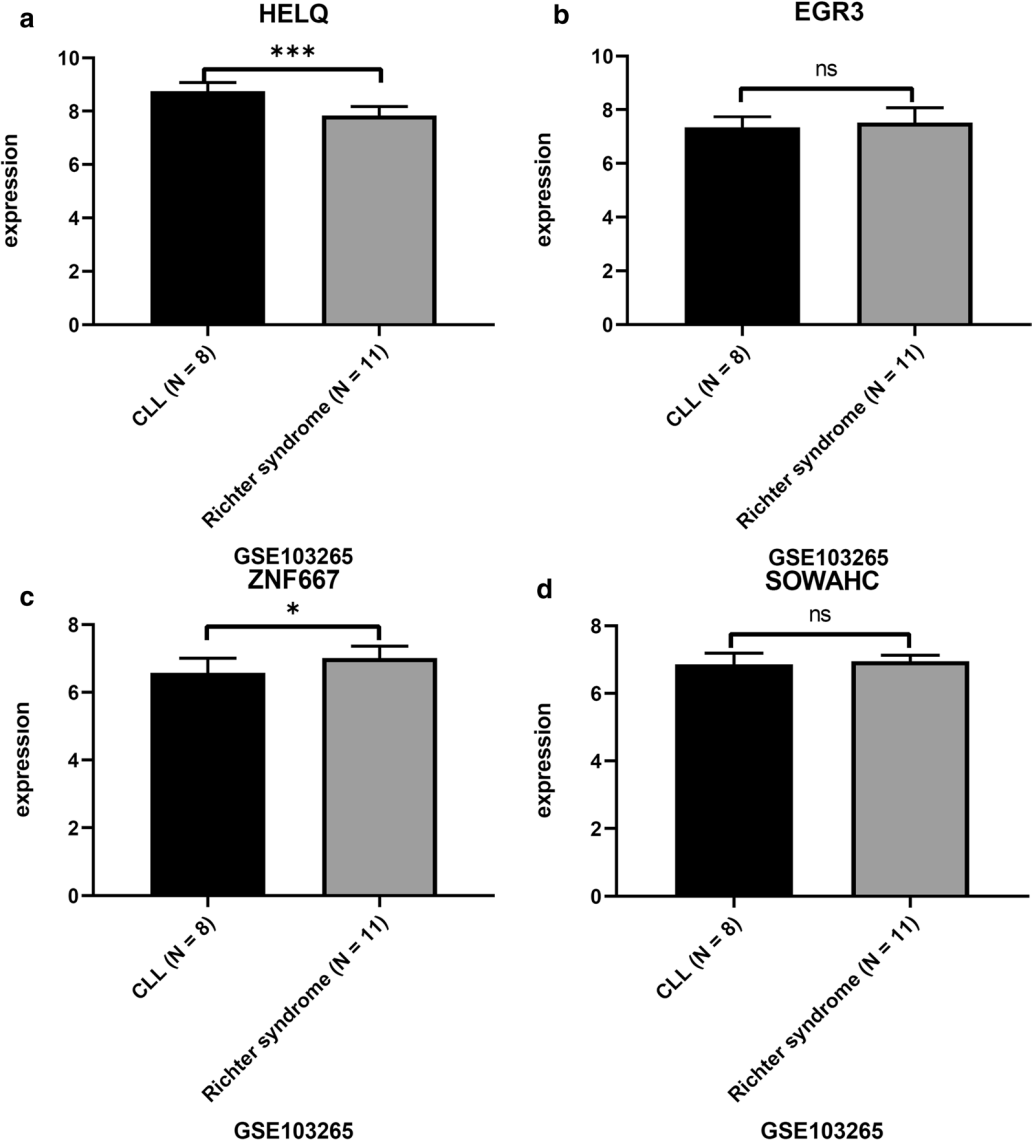
The comparison of hub genes expression between Richter transformed and non-transformed CLL based on GSE103265. ***p < 0.0001. *p < 0.05. ns, p > 0.05

No significant association between the Binet clinical stage and expression of all hub genes, was uncovered in our analysis based on GSE58211 (data not shown). The higher expression of HELQ predicted stable disease instead of progressive disease for CLL in GSE10138 (Fig. 7a, p = 0.0413), while other hub genes were not significantly expressed differently (Fig. 7b–d). In Richter transformed CLL patients, the expression of HELQ significantly decreased (p < 0.0001), while the expression of ZNF667 significantly increased (p = 0.0278) in comparison with non-transformed cases (Fig. 8).

### HELQ and EGR3 predicted TTFT and OS for CLL patients

**Fig. 9:**
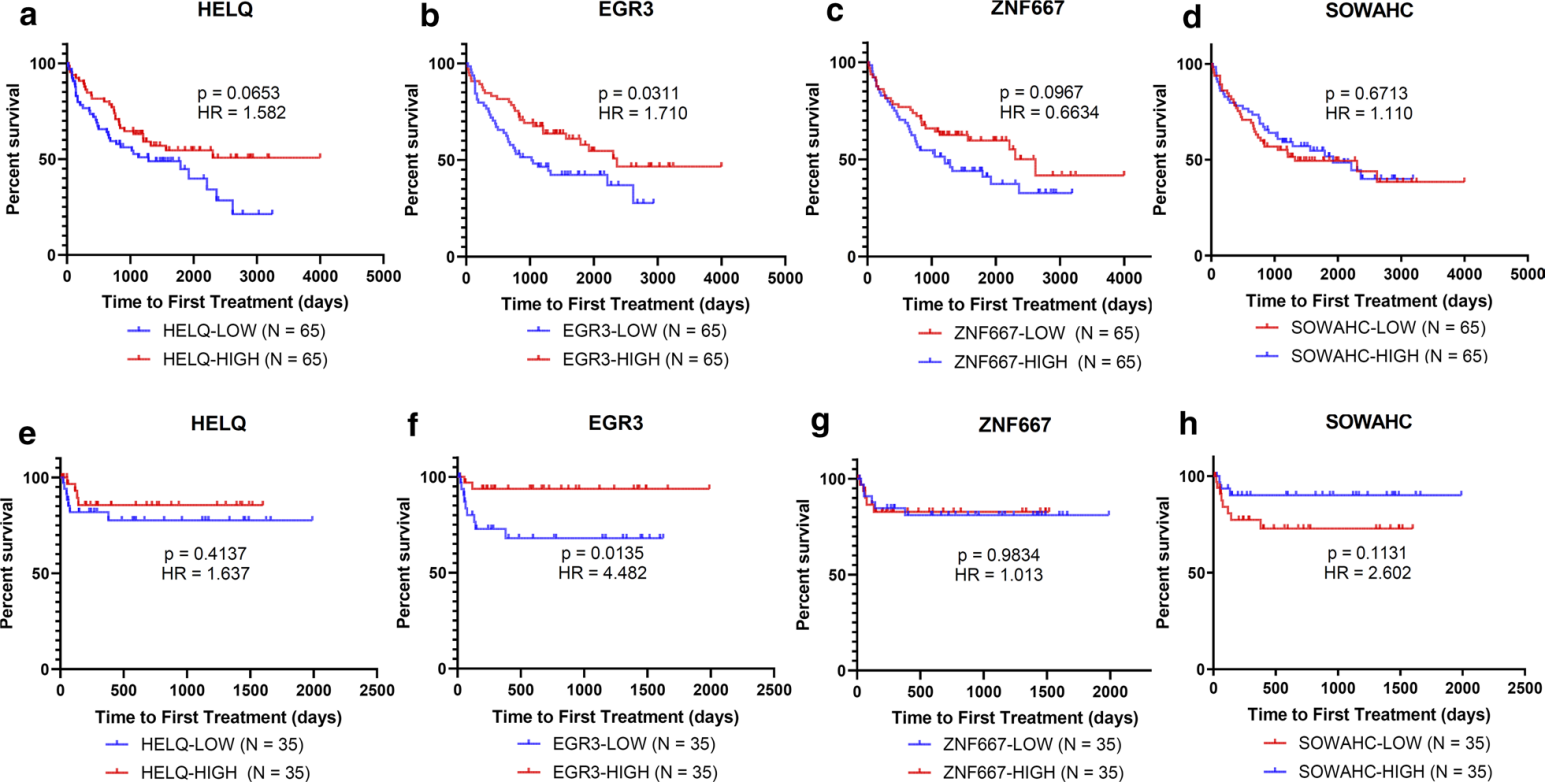
Kaplan–Meier plots for TTFT of subgroups dichotomized by hub genes expression, based on GSE39671 and GSE22762

The analysis on impact of hub genes expression on TTFT was shown in Fig. 9. EGR3-low group had significantly shorter TTFT in comparison with EGR3-high group based on GSE39671 (Fig. 9b) and GSE22762 (Fig. 9f). Although it’s not significant, the TTFT of HELQ-low group tended to be inferior to HELQ-high group (Fig. 9a, e). No significantly results of TTFT analysis were found for ZNF667 (Fig. 9c, g) and SOWAHC (Fig. 9d, h), either. However, intriguing trends were revealed for ZNF667 (Fig. 9c) and SOWAHC (Fig. 9h), which demonstrated that the survival of subgroups was not significantly different (p > 0.05), but the Kaplan–Meier curves were separated (Fig. 9c, h).

**Fig. 10:**
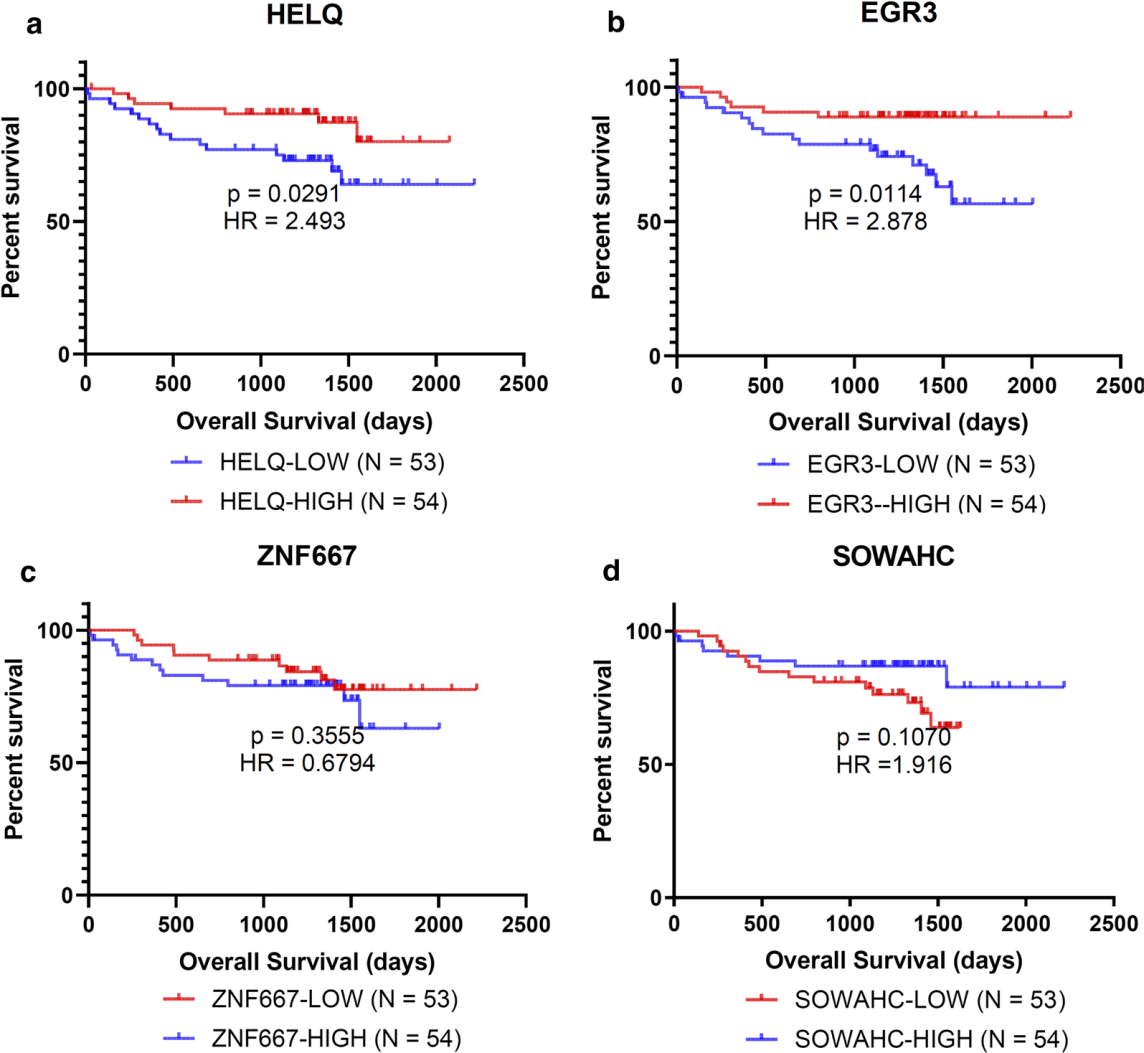
Kaplan–Meier plots for OS of subgroups dichotomized by hub genes expression, based on GSE22762

The survival analysis using GSE22762 dataset was shown in Fig. 10. The HELQ-low group showed significantly unfavorable OS in comparison with the HELQ-high group (Fig. 10a). The OS of the EGR3-low group was also inferior over the EGR3-high group (Fig. 10b).

### The results of GSEA for HELQ and EGR3

**Fig. 11:**
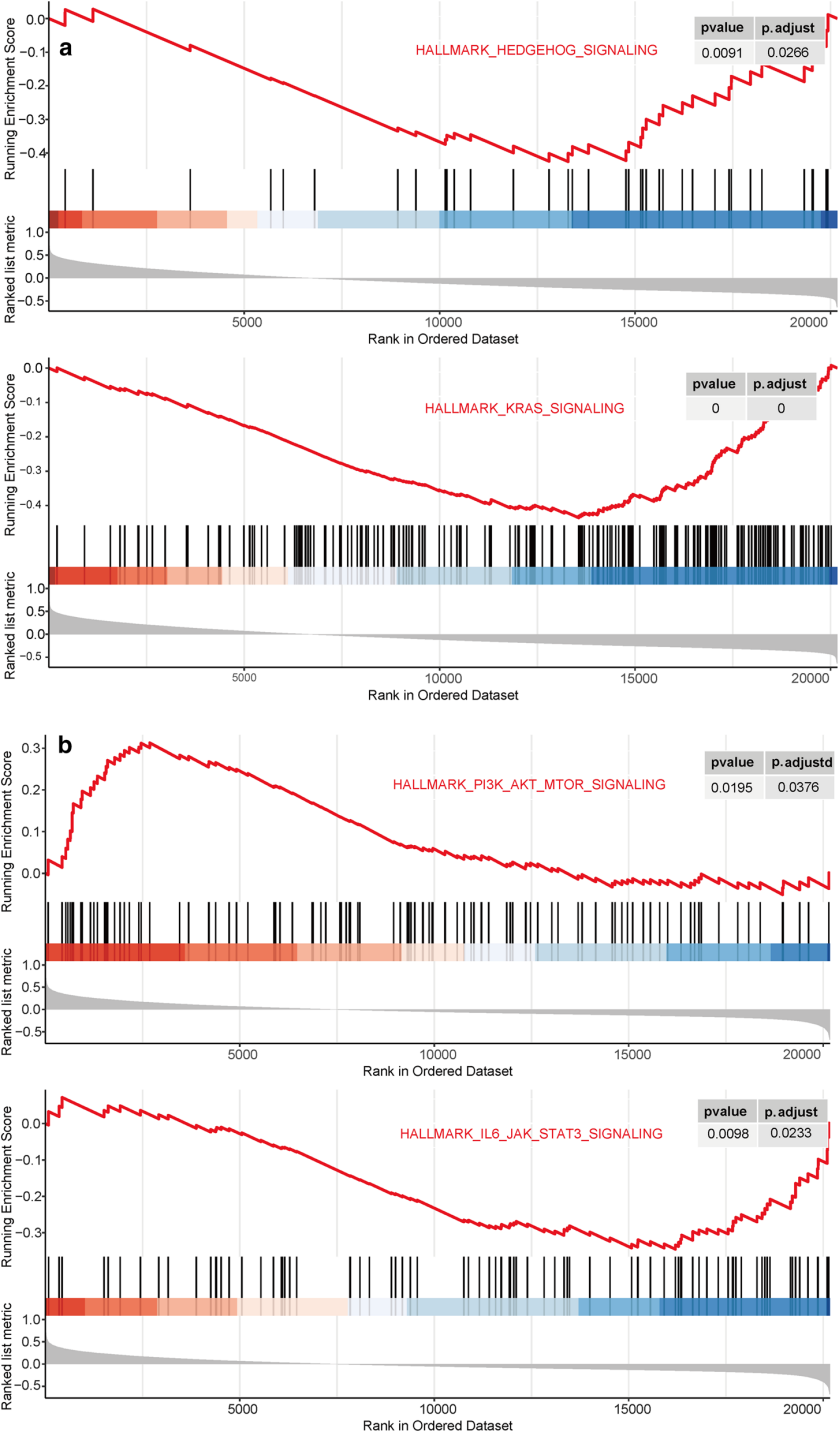
The running enrichment score curve for pathways significantly correlating with HELQ (**a**) and EGR3 (**b**)

The results of GSEA were shown in Table 3. The activated pathways associating with HELQ expression, included MYC (Myc proto-oncogene protein) targets, E2F (Transcription factor E2F1 targets and DNA repair pathways, etc. while the suppressed pathways associating with HELQ expression, included Hedgehog signaling, Kras signaling pathway etc. (Fig. 11a). The activated pathways correlating with EGR3 expression, included MYC targets, E2F targets, PI3K-Akt-mTOR signaling pathways, etc. while the suppressed pathways correlating with EGR3 expression, included IL6-JAK-STAT3 signaling, Kras signaling pathway etc. (Fig. 11b).

**Table 3:** The results of GSEA for HELQ and EGR3 with significantly enriched pathways

	Pathway	Enrichment Score	NES	P value	FDR adjusted p value	Q values
HELQ expression associated pathways	Kras signaling	− 0.435668418	− 2.1532	1.00E−10	5.00E−09	3.05E−09
	Hedgehog signaling	− 0.42542167	− 1.6012	0.00905	0.0266321	0.01626
	coagulation	− 0.40041732	− 1.9035	1.78E−06	1.78E−05	1.09E−05
	Myogenesis	− 0.366088795	− 1.8159	3.62E−06	2.64E−05	1.61E−05
	Epithelial mesenchymal transition	− 0.365397776	− 1.8159	3.69E−06	2.64E−05	1.61E−05
	Bile acid metabolism	− 0.340839788	− 1.5737	0.00346	0.0123508	0.00754
	Estrogen response late	− 0.335392317	− 1.6646	7.44E−05	0.0004131	0.00025
	Inflammatory response	− 0.332882791	− 1.6521	0.0001	0.000507	0.00031
	Spermatogenesis	− 0.312304664	− 1.4807	0.00614	0.0191922	0.01172
	Estrogen response early	− 0.307378963	− 1.5247	0.00124	0.0051754	0.00316
	Xenobiotic metabolism	− 0.304376309	− 1.5098	0.00158	0.0060767	0.00371
	DNA repair	0.240957608	1.47711	0.00535	0.0178322	0.01089
	MYC targets	0.255275512	1.66642	7.34E−05	0.0004131	0.00025
	G2M checkpoint	0.301740945	1.98069	1.82E−07	2.28E−06	1.39E−06
	E2F targets	0.304239585	1.99709	1.16E−07	1.93E−06	1.18E−06
	Mitotic spindle	0.30860828	1.97486	4.61E−08	1.15E−06	7.04E−07
	Protein secretion	0.32775742	1.88719	0.00013	0.0005808	0.00035
EGR3 expression associated pathways	Interferon alpha response	− 0.498828541	− 2.3019	7.24E−09	3.62E−07	1.45E−07
	Cholesterol homeostasis	− 0.433630867	− 1.8937	8.29E−05	0.0003453	0.00014
	Reactive oxigen species pathway	− 0.410797211	− 1.6669	0.00434	0.0120436	0.00482
	Coagulation	− 0.381436147	− 1.8811	8.40E−06	5.59E−05	2.24E−05
	Myogenesis	− 0.35817246	− 1.8869	3.24E−06	3.24E−05	1.30E−05
	Interferon gamma response	− 0.353929759	− 1.8643	1.82E−06	2.27E−05	9.09E−06
	IL6-JAK-STAT3 signaling	− 0.345542037	− 1.5649	0.00978	0.0232876	0.00932
	Kras signaling	− 0.329603523	− 1.732	8.04E−05	0.0003453	0.00014
	epithelial mesenchymal transition	− 0.32757194	− 1.7247	4.34E−05	0.0002168	8.67E−05
	Estrogen response late	− 0.321965048	− 1.6931	0.00013	0.0004868	0.00019
	Inflammatory response	− 0.306318547	− 1.6109	0.00055	0.0019486	0.00078
	Complement	− 0.286012557	− 1.5067	0.00293	0.0086057	0.00344
	Apoptosis	− 0.278729142	− 1.4182	0.01129	0.0243914	0.00976
	Estrogen response early	− 0.272180964	− 1.4339	0.00861	0.0215358	0.00861
	Glycolysis	− 0.268495638	− 1.4144	0.01088	0.0243914	0.00976
	Adipogenesis	− 0.26708006	− 1.4058	0.00807	0.0212352	0.00849
	Hypoxia	− 0.264798339	− 1.3925	0.01171	0.0243914	0.00976
	Allograft rejection	− 0.260668803	− 1.3732	0.01705	0.0340906	0.01364
	PI3K-Akt-mTOR signaling	0.312772587	1.4756	0.01953	0.0375598	0.01502
	DNA repair	0.326826349	1.62277	0.00084	0.0027889	0.00112
	Mitotic spindle	0.339594124	1.78072	3.16E−05	0.0001753	7.01E−05
	G2M checkpoint	0.357870462	1.86809	4.20E−06	3.50E−05	1.40E−05
	E2F targets	0.399460879	2.08519	2.71E−08	4.51E−07	1.80E−07
	MYC targets v1	0.401746954	2.09159	1.97E−08	4.51E−07	1.80E−07
	MYC targets v2	0.428081704	1.80452	0.00173	0.0054071	0.00216
	Protein secretion	0.440075051	2.06176	8.94E−06	5.59E−05	2.24E−05

The associated pathways of HELQ and EGR3 were overlapped, among which E2F signaling, MYC signaling and DNA repair pathways were activated in both sets, whereas Kras signaling/inflammatory response were both suppressed.

## Discussion

The expression signature of patients harboring different status of IGHV mutation, has been explored previously [35–38]. But no researchers investigated the co-expression modules correlating with IGHV status using WGCNA. WGCNA is a clustering method to investigate the scale-free property of gene expression network, in which TOM was used to evaluate the connectivity of individual genes [39]. Then the co-expression modules were identified, genes of which is highly co-expressed and strongly connected in network[40].

In the results of WGCNA, the ‘black’ module was significantly correlated with M-CLL, and the ‘purple’ module was significantly correlated with U-CLL. Notably, the 2 modules were connected in the protein–protein interaction analysis based on pre-existing experimental evidence in STRING database (Additional file 2: Fig. S2). Moreover, ORA revealed several overlapped pathways enriched by both modules, such as mTOR, Hippo and p53 signaling (Fig. 3). In accordance with our results, mTOR signaling was activated in the majority of CLL patients, but in 2 different manners [41]. In most of U-CLL, mTOR activation occurs downstream of BCR signaling. While a subset of M-CLL is driven by mTOR signaling in a non-BCR-dependent manner.

The ORA based on GO database demonstrated that genes in the ‘black’ module were mainly enriched in biological processes related with B cell activation/proliferation/apoptosis. Of note, negative regulation of B cell receptor signaling were found to be enriched by gene set of the ‘black’ module (p = 0.00185), which involved PLCL2 and FCRL3. PLCL2 expresses in hematopoietic cells, encoding phospholipase C-L2 protein. The B cells in PLCL2-knockout mice were highly proliferative to cross-linking of BCR signaling [42], suggesting a negative regulation role in BCR signaling. FCRL3, encoding Fc receptor-like protein 3, is reported to be highly expressed in M-CLL instead of U-CLL [43], which demonstrates the inhibitory potential on BCR signaling [44]. And deletion or downregulation of FCRL3 predicts poor prognosis for CLL patients [45].

For the ‘black’ module, ORA according to KEGG database, demonstrated the NF-kappaB, HIF-1 and AMPK signaling pathways were enriched for the ‘black’ module. The activity of NF-kappaB signaling is demonstrated to be variable but overall increased in CLL leukemic cells [46–48], which is also crucial for survival of leukemic cells [47] and potentially targetable for CLL [49]. HIF-1a expression and HIF-1 signaling is demonstrated to promote the interaction of CLL leukemic cells and microenvironment [51], which facilitates the survival and propagation of CLL. The transcription of HIF-1 is increased in TP53-disrupted CLL patients, while the HIF-1 induced interaction, between leukemic cells and stromal cells, is independent of TP53 status [52]. The inhibition of HIF-1 signaling is potentially therapeutic, especially in M-CLL based on our analysis. The previous report indicates AMPK signaling is in the control of apoptosis for CLL cells [53, 54], which can be activated by acadesine in a p53 independent way. Additionally, AMPK signaling can be activated by ATP deleting agents, such as metformin and 8-chloro-adenosine, which will switch the energy-generating pathways, leading to autophagy both in vitro and in vivo [55, 56]. Therefore, the AMPK signaling and autophagy are potential crucial pathways in M-CLL.

In ORA for the ‘purple’ module, Wnt signaling was enriched for U-CLL patients. The Wnt signaling is required for survival of CLL leukemic cells in the functional study [58], which is activated by somatic mutations [59] or Notch2 activity from BMSC [60]. Combining with our ORA analysis for the ‘purple’ module, inhibition of Wnt signaling or Notch 2 was potential therapeutic for U-CLL patients.

A total of 9 hub genes were identified by WGCNA, including FCRL1, FCRL2, HELQ, EGR3, LPL, LDOC1, ZNF667, SOWAHC and SEPTIN10. The impact of Fc receptor like molecules (including FCRL1 and FCRL2) have been elucidated in CLL, which predicts the IGHV mutation status and clinical progression [43], and can act as potential immunotherapeutic targets [61, 62]. LPL, encoding lipoprotein lipase, is expressed in CLL patients with aggressive clinical properties, which promotes activating ligands for PPARα (Peroxisome proliferator-activated receptor alpha) and switch energy source to fatty acid [63]. Additionally, the LPL expression is reported to be a strong prognostic indicator [64]. Hatice Duzkale et al. demonstrate that mRNA expression of LDOC1 is correlated with prognostic markers (cytogenetic markers, IGHV mutation status, and ZAP-70 expression), also a predictor of OS for CLL patients [65]. The expression of SEPTIN10 is reported to an independent prognostic factor for survival [66] and TTFT [37] of CLL patients. 4 other genes were not reported in previous studies, and selected as target genes in our study, due to the prognostic value in cancers and correlation with IGHV status.

The correlation of hub genes expression with IGHV status in external cohorts was consistent with the result obtained from WGCNA (Fig. 4, Table 2), in which the HELQ and EGR3 were overexpressed in M-CLL than that of U-CLL significantly, while ZNF667 and SOWAHC were under-expressed.. The differential expression was found to be significant for HELQ and ZNF667 between CLL cells and normal B cells (Fig. 5). Moreover, HELQ, EGR3 and ZNF667 were expressed differentially among diverse types of lymphoid neoplasm (Fig. 6). The above results suggested that the expression signature of hub genes is CLL-specific.

The analysis on GSE10138 indicated higher expression of HELQ correlated with better response to immunochemotherapy (Fig. 7). Additionally, the results obtained from GSE103265 suggested that HELQ and ZNF may serve as potential indicators for Richter transformation (Fig. 8), which may help to predict high risk CLL patients. The association of hub genes expression with clinical features suggested that these genes may involve in pathogenesis of CLL.

As the individual course of early stage CLL is heterogenous, the anticipation for urgency and probability of more aggressive intervention is still not solved. Our results demonstrated that EGR3-high group had significant longer TTFT and more indolent disease course, which were consistent with International Prognostic Score for Early-stage CLL (IPS-E) system[67], in which U-CLL predicts unfavorable TTFT. Furthermore, our analysis on OS confirmed the survival advantage of HELQ-high as well as EGR3-high group (GSE22762). Although novel target agents have greatly improved the prognosis of CLL patients than traditional immunochemotherapy, the prognostic value of HELQ and EGR3 is still potentially crucial, the re-evaluation of which is worthy to conduct in the novel drug era.

Due to the relevance of HELQ and EGR3 with disease course and prognosis of CLL, we performed GSEA to reveal the significantly activated and/or suppressed signaling pathways correlating with HELQ/EGR3 overexpression (Fig. 11 and Table 3). The GSEA indicated that Kras signaling and Hedgehog signaling was negatively correlated with HELQ expression. The somatic mutations in RAS signaling pathway, including KRAS, occurs in a subset of CLL cases, who more frequently have unmutated IGHV gene and worse TTFT [68]. While the functional activation, instead of activating mutations, of Kras signaling has not been reported yet. The inhibitors of Kras signaling, such as ulixertinib [68], may provide a new treatment option for HELQ-low or U-CLL patients. The Hedgehog signaling is implicated in the initiation, maintenance [69], and survival [70] of CLL cells. CLL patients with activation of Hedgehog pathway are associated with a shorter median TTFT [71], which may attribute to the inferior clinical outcomes in HELQ-low group. Although vismodegib, a Hedgehog inhibitor, significantly suppressed the Hedgehog signaling in CLL patients, no patients response to vismodegib treatment in a phase II clinical trials [72]. Limited number of included patients and ‘ligand-independent’ activation of Hedgehog pathway may contribute to the failure.

The IL6-JAK-STAT3 signaling was also demonstrated to be negatively correlated with EGR3 expression (Table 3). In CLL cells, extracellular IL6 or BCR signaling induces tyrosine phosphorylation of STAT3 [73, 74], leading to upregulated of anti-apoptosis genes and a survival advantage. The BMSC are reported to interact with CLL cells by modulating JAK2/STAT3 signaling [75], protecting from CLL cells from ibrutinib. This effect can be reversed by combination of ibrutinib and JAK2 inhibitor (AG490), which triggers apoptosis of CLL cells even in the presence of BMSC. Therefore, additional inhibition of IL6-JAK-STAT pathway may be a potential option for EGR3-low or U-CLL patients, which facilitate the clearance of residual CLL cells in protective bone marrow microenvironment after ibrutinib treatment. The PI3K-Akt-mTOR signaling was positively correlated with EGR3 expression. According to the DNA perturbation based stratification study, signaling mediated via mTOR plays a greater role than canonical BCR signaling for survival/proliferation of a M-CLL subset, as the effect of inhibiting mTOR is greater than BTK [41]. So, for M-CLL patients, high expression of EGR3 may be an indicator for usage of additional mTOR inhibitor.

The MYC target signaling was activated in both HELQ-high and EGR3-high group (Table 3). MYC and downstream targets play a role in antigen induced CLL proliferation [76, 77]. The GSEA indicated the possible pathways related with HELQ/EGR3 expression and provided insights to investigation of personalized therapy.

## Conclusion

We identified the co-expression modules and hub genes correlating with IGHV mutation status in CLL patients. The differential expression of hub genes was validated by external cohorts, and associated with clinical features like treatment response and Richter transformation. HELQ and EGR3 expression were prognostic markers and predicted TTFT and OS.

## Supplementary Information

**Additional file 1: Figure S1.** The plot of sample clustering to detect outliers for GSE22654.

**Additional file 2: Figure S2.** The protein-protein interaction network for the ‘black’ and ‘purple’ modules. The color of node symbol is black for genes from the ‘black’ module, as well as purple symbol for genes from the ‘purple’ module. The color of node indicated the connectivity degrees (red for high degrees, blue for low degrees).

## Data Availability

The data that support the findings of this study are available from GEO database (https://www.ncbi.nlm.nih.gov/gds/), which are all publicly available.

## Abbreviations

CLL: Chronic lymphocytic leukemia
IGHV: Immunoglobulin heavy-chain variable region
WGCNA: Weighed gene co-expression network analysis
OS: Overall survival
TTFT: Time to first treatment
GSEA: Gene set enrichment analysis
M-CLL: IGHV gene mutated CLL
U-CLL: IGHV gene unmutated
BCR: B cell signaling
BTK: Bruton's Tyrosine Kinase
BCL2: B cell lymphoma 2
FCRL1: Fc receptor-like protein 1
FCRL2: Fc receptor-like protein 2
HELQ: Helicase POLQ-like
EGR3: Early growth response protein 3
LPL: Lipoprotein lipase
LDOC1: Protein LDOC1
ZNF667: Zinc finger protein 667
SOWAHC: Ankyrin repeat domain-containing protein SOWAHC
SEPTIN10: Septin-10
GEO: Gene expression omnibus
TOM: Topological overlap matrix
ME: Modules eigengene
FL: Follicular lymphomas
DLBCL: Diffuse large B cell lymphoma
MCL: Mantle cell lymphomas
MALT: Mucosa associating lymphoid tissue
MZL: Marginal zone lymphoma
NMZL: Nodal marginal zone lymphoma
STRING: Search Tool for the Retrieval of Interacting Genes/Proteins
ORA: Over-representation analysis
PPI: Protein–protein interaction
GO: Gene ontology
KEGG: Kyoto Encyclopedia of Genes and Genomes
NES: Normalized enrichment score
FDR: False discovery rate
PPAR: Peroxisome proliferator activated receptor
MYC: Myc proto-oncogene protein
