# Supplementary figures and images for: HELQ and EGR3 expression correlate with IGHV mutation status and prognosis in chronic lymphocytic leukemia
Source: J Transl Med. 2021 Jan 23;19:42. doi: 10.1186/s12967-021-02708-6 (PMC7825181; doi:10.1186/s12967-021-02708-6)

# Sample clustering to detect outliers

Height

50 70 90 110

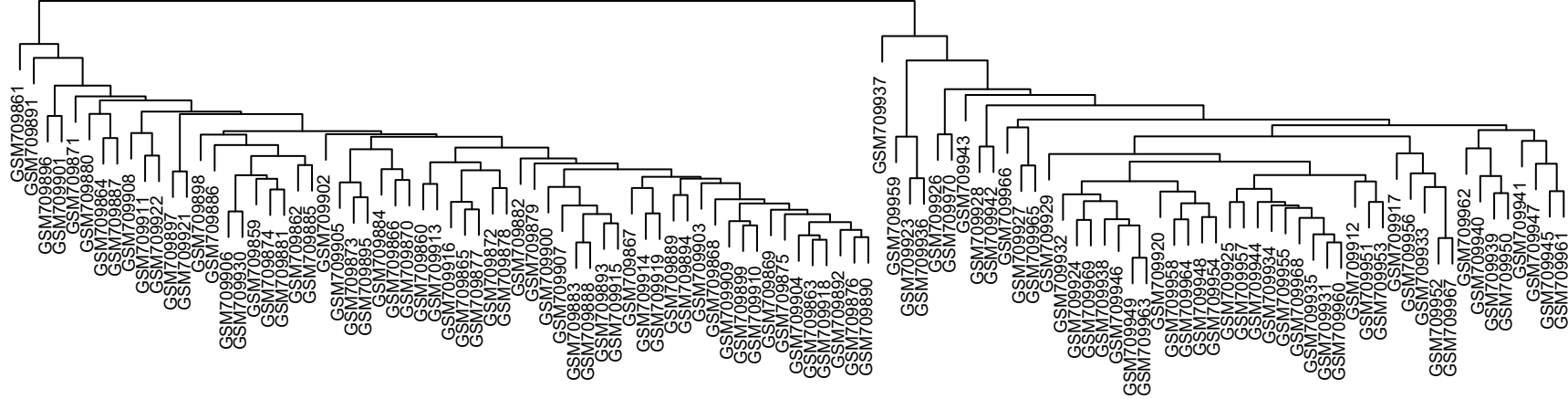

Supplement: Supplementary file 1 — Additional file 1: Figure S1. The plot of sample clustering to detect outliers for GSE22654. [file 12967_2021_2708_MOESM1_ESM.pdf]

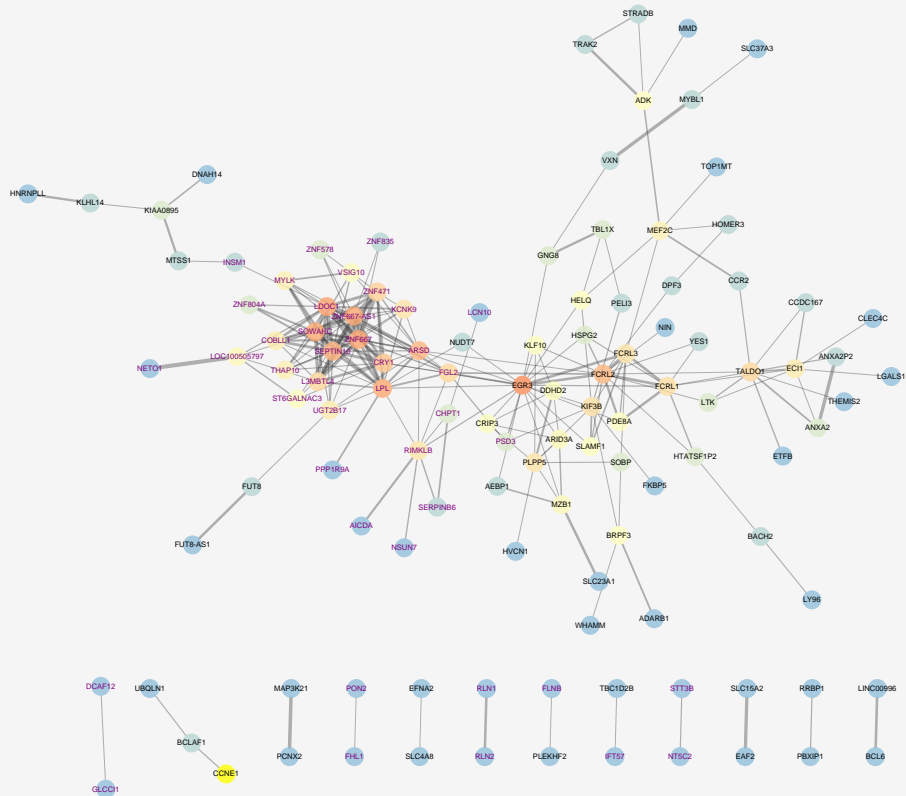

Supplement: Supplementary file 2 — Additional file 2: Figure S2. The protein-protein interaction network for the ‘black’ and ‘purple’ modules. The color of node symbol is black for genes from the ‘black’ module, as well as purple symbol for genes from the ‘purple’ module. The color of node indicated the connectivity degrees (red for high degrees, blue for low degrees). [file 12967_2021_2708_MOESM2_ESM.pdf]
